# Patient-level comparison of heart failure patients in clinical phenotype and prognosis from China and Sweden

**DOI:** 10.1186/s12872-022-02540-w

**Published:** 2022-03-08

**Authors:** Yizhou Feng, Xiaojing Chen, Maria Schaufelberger, Qing Zhang, Michael Fu

**Affiliations:** 1 Department of Cardiology, West China Hospital, Sichuan University, Chengdu, 610041 Sichuan People’s Republic of China; 2 Department of Molecular and Clinical Medicine, Institute of Medicine, Sahlgrenska Academy, University of Gothenburg, Gothenburg, Sweden

**Keywords:** Heart failure, China, Sweden, Therapy, Prognosis

## Abstract

**Background:**

Clinical phenotype and prognosis of heart failure (HF) may be variable among different racial populations. Therefore, a patient-level comparison of hospitalized HF patients in two university hospitals from China and Sweden was performed.

**Methods and results:**

This study was a pooled data analysis of the patients prospectively enrolled in two single-center studies in China (n = 949) and Sweden (n = 1639) from 2011 to 2015. Clinical characteristics and 6-month all-cause mortality were collected. Higher systolic blood pressure (126.1 ± 20.3 vs. 114.2 ± 15.4 mmHg, p < 0.001) and NT-proBNP level (4540 vs. 3251 pg/mL, p = 0.013) were found in the Swedish cohort, also more patients with ischemic heart disease (32.0% vs. 19.2%), hypertension (64.2% vs. 36.8%), valvular heart disease (40.9% vs.31.6%) and atrial fibrillation (55.3% vs. 39.6%) (all p < 0.001). The use of ACEIs/ARBs (48.8% vs. 80.8%) or beta-blockers (58.8% vs. 86.5%) (both p < 0.001) was lower in Chinese cohort. Given younger age in Chinese cohort (61.6 vs. 76.4 years, p < 0.001), age-stratified analyses were conducted, as there were similar patient numbers in 50–74 years in Chinese (n = 550) and Swedish (n = 554) cohorts, therefore baseline characteristics and prognosis were further compared. The age- and sex-adjusted outcome (HR 0.80 [95% CI 0.55–1.19], p = 0.273) was comparable between the two populations. The NT-proBNP and eGFR independently predicted 6-month mortality in both Chinese (HR [95% CI] 1.006 [1.003–1.008], 0.986 [0.976–0.999]) and Swedish cohort (1.003 [1.000–1.007], 0.988 [0.976–0.999]).

**Conclusions:**

Patient-level comparison of real-world HF populations from China and Sweden demonstrated different clinical phenotypes and therapy but similar prognosis and their predictors.

**Supplementary Information:**

The online version contains supplementary material available at 10.1186/s12872-022-02540-w.

## Introduction

Heart failure (HF) is a major worldwide health problem with a prevalence estimated to 0.9% in China [1] and even higher in European countries (1.5–2%) [2]. Although guideline-directed therapy has now been proven to reduce mortality and morbidity [3], HF remains the leading cause of hospitalization with a rate of death varying from about 10% after 1 year to about 50% after 5 years from diagnosis [4]. Therefore, it is a global public health concern and places a significant economic burden on the health care system in both developed and developing countries [5, 6].

Epidemiology, clinical profile, management and prognosis of HF have been well described in a number of clinical trials [7–10] and large registries [11–16] performed in developed countries like North America and Europe, however, there is limited information derived from the Chinese HF population [17–22]. Few data have suggested regional and ethnic heterogeneity between China and Western countries [23], and to what extent such discrepancies contribute to prognosis of HF patients in a real-world clinical setting remains largely unexplored. Therefore, we compared hospitalized HF patients of two single-center registries from China and Sweden, with regard to clinical characteristics, HF therapy and prognosis.

## Methods

### Study design, setting and populations

This study was a pooled data analysis of two different ethnic groups. The study population consisted of patients from two HF registries, one in China and the other in Sweden. The former was conducted in a large tertiary referral hospital in China (West China Hospital, Sichuan University, Chengdu), while the latter was established in a leading hospital (Sahlgrenska University Hospital/Östra Hospital, Sweden) of the Swedish Heart Failure Registry (SwedeHF) [24]. The two registries complied with the 2008 Declaration of Helsinki and were approved by the Chinese Ethics Committee of Registering Clinical Trials (West China Hospital) and the Ethics Committees of the University of Gothenburg. Informed consent was obtained from all participants or, if participants were dead, from a next of kin and/or legal guardian. Patients aged ≥ 18 years old hospitalized for HF were enrolled in the registries according to the diagnostic criteria of the guidelines prevailing at that time, without specific exclusion criteria. Only those who underwent coronary artery revascularization and valve intervention during the indexed hospitalization failed to be included in the Chinese registry. Both registries recorded variables regarding patient characteristics and therapy at discharge and prognosis at follow-up. As the overlapping period of enrollment in the two registries was from December, 2011 to December, 2015, patients registered within that time were selected for the current study.

### Data collection

Variables included in both cohorts were matched based on unified definitions according to existing guidelines at the time of patient enrollment. Variables with more than 25% missing values were excluded.

The study population was divided into two subgroups with left ventricular ejection fraction (LVEF) either < 40% (HFrEF) or ≥ 40% (non-HFrEF) due to two reasons: 1) only in those with LVEF < 40% there are evidence-based lifesaving therapy, and 2) there was no uniform definition of those patients with HF with LVEF ≥ 40% during the study period. When the study was initiated in 2011, a preserved left ventricular ejection fraction (LVEF) was defined as > 40–50% [25] until 2012 thereafter LVEF was set to ≥ 50% and renamed uniformly as HFpEF [26]. Blood pressure was recorded in the right arm in the sitting position by a mercury sphygmomanometer, and heart rate was measured by 12-lead electrocardiography (ECG) in the supine position. Anemia was defined as hemoglobin level < 12 g/dL in women and < 13 g/dL in men [27]. The Chronic Kidney Disease Epidemiology Collaboration (CKD-EPI) equation was used to calculate the estimated glomerular filtration rate (eGFR), while renal dysfunction was defined as eGFR ≤ 60 ml/min/1.73m^2^ [28].

### Follow up and end-points

All patients were followed-up for at least 6 months, and the primary outcome measure was 6-month all-cause mortality. It was obtained by phone call and/or review of the medical record in the Chinese cohort, whereas by the automatic linking of SwedeHF with the Cause of Death Registry of the National Board of Health and Welfare of Sweden every month in the Swedish cohort.

### Statistical analyses

Normally distributed continuous data are presented as mean ± standard deviation (SD), whereas non-normally distributed variables are presented as median with interquartile ranges. Categorical data are presented as numbers and percentages. Comparisons between the two cohorts were performed by the independent-sample *t* test or Mann–Whitney U-test for continuous variables and the chi-square test for categorical variables. Missing data were handled by multiple imputation (n = 10), respectively in the two cohorts. Given a different age distribution between the two cohorts, age-stratified analyses of prognosis were conducted. Kaplan–Meier curves and log-rank tests were used to compare survival and Cox proportional models to explore the predictors of mortality. Statistics were performed using IBM SPSS Statistics 25.0 (IBM Corp, Armonk, NY, USA). A p value of < 0.05 (two tailed) was considered statistically significant.

## Results

**Fig. 1:**
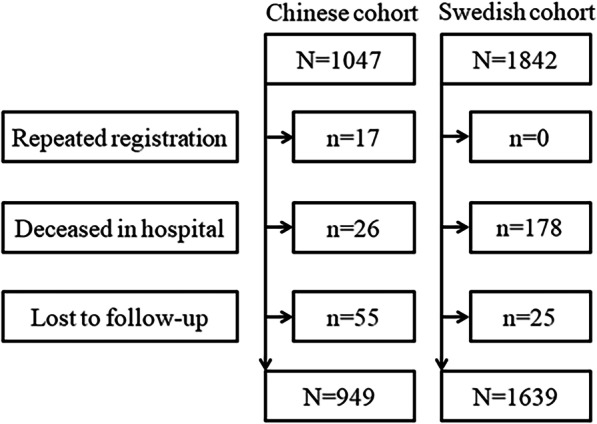
Flow chart of the patient enrollment

Between 2011 and 2015, 1047 patients from West China Hospital and 1842 patients from Sahlgrenska University Hospital/Östra Hospital were screened for eligibility of pooled analysis. Figure 1 depicts the enrollment. Finally, 949 patients in the Chinese cohort and 1639 patients in the Swedish cohort were studied.

### Clinical phenotype

Patients in the Chinese cohort were younger (Median 64 [IQR] 52–73 vs. 79 [IQR] 69–86 years in the Swedish cohort, p < 0.001) (Table 1). Proportion of HFrEF (40.8% vs. 44.1%, p = 0.084) and non-HFrEF was similar in Chinese and Swedish cohort. Systolic (126.1 ± 20.3 vs. 114.2 ± 15.4 mmHg, p < 0.001) and diastolic (71.7 ± 11.3 vs 69.1 ± 10.1 mmHg, p < 0.001) blood pressure were higher in the Swedish patients, whereas heart rate was lower (72.2 + 14.3 vs.83.7 ± 20.9 beats/min, p < 0.001). N-termin pro-brain natriuretic peptide (NT-proBNP) level was higher in the Swedish cohort (Median 4540 vs. 3251 pg/mL, p = 0.013).

**Table 1:** Baseline characteristics of patients in the two cohorts

Variables	Chinese cohort (N = 949)	Swedish cohort (N = 1639)	P-value
*Demographics*			
Age, years			
Mean (SD)	61.6 ± 15.0	76.4 ± 13.4	**< 0.001**
Median (IQR)	64 (52–73)	79 (69–86)	**< 0.001**
Female, n (%)	442 (46.6)	677 (41.6)	**0.013**
*Clinical history, n (%)*			
Ischemic heart disease	182 (19.2)	524 (32.0)	**< 0.001**
Dilated cardiomyopathy	245 (25.8)	116 (7.1)	**< 0.001**
Hypertension	349 (36.8)	1050 (64.2)	**< 0.001**
Valvular disease	300 (31.6)	671 (40.9)	**< 0.001**
Atrial fibrillation/flutter	376 (39.6)	905 (55.3)	**< 0.001**
Diabetes mellitus	295 (31.1)	465 (28.4)	0.144
Pulmonary disease	118 (12.4)	352 (21.5)	**< 0.001**
Anemia	359 (37.9)	708 (43.5)	**0.005**
*Physical/laboratory*			
SBP, mmHg	114.2 ± 15.4	126.1 ± 20.3	**< 0.001**
DBP, mmHg	69.1 ± 10.1	71.7 ± 11.3	**< 0.001**
Heart rate, beats/min	83.7 ± 20.9	72.2 ± 14.3	**< 0.001**
Hemoglobin, g/L	129.3 ± 23.9	127.8 ± 18.0	**0.002**
eGFR, ml/min/1.73m^2^	63.5 ± 32.4	61.6 ± 35.8	**0.002**
NT-proBNP,	3251	4540	**0.013**
Median (IQR), pg/mL	(1469–7602)	(1750–9475)	
LVEF, %, n (%)			0.084
≥ 40	562 (59.4)	916 (55.9)	
< 40	387 (40.8)	723 (44.1)	
QRS duration, ms, n (%)			**< 0.001**
≥ 120	288 (30.3)	605 (36.9)	
< 120	661 (69.7)	1034 (63.1)	
LBBB, n (%)	174 (18.4)	148 (10.0)	**< 0.001**
*Medication, n (%)*			
ACEIs/ARBs	463 (48.8)	1324 (80.8)	**< 0.001**
Beta-blockers	558 (58.8)	1417 (86.5)	**< 0.001**
Aldosterone antagonists	632 (66.6)	502 (30.6)	**< 0.001**
Diuretics	724 (76.3)	1232 (75.2)	0.522
Digitalis	379 (39.9)	215 (13.1)	**< 0.001**
*Device therapy, n (%)*			
Pacemaker	47 (5.0)	193 (11.8)	**< 0.001**
CRT-P/D	57 (6.0)	35 (2.1)	**< 0.001**
ICD	44 (4.6)	24 (1.5)	**< 0.001**

SD, standard deviation; IQR, interquartile range; S/DBP, systolic/diastolic blood pressure; eGFR, estimated glomerular filtration rate; NT-proBNP, N-terminal pro brain natriuretic peptide; LVEF, left ventricular ejection fraction; LBBB, left bundle branch block; ACEI, angiotensin-converting enzyme inhibitor; ARB, angiotensin receptor blocker; CRT-P/D, cardiac resynchronization therapy with pacemaker/defibrillation; ICD, implantable cardioverter defibrillator

Comorbidities were significantly different between the two cohorts. In the Swedish cohort there were more patients with ischemic heart disease (32.0% vs. 19.2%, p < 0.001), hypertension (64.2% vs. 36.8%, p < 0.001), valvular heart disease (40.9% vs.31.6%, p < 0.001), atrial fibrillation/flutter (55.3% vs.39.6%, p < 0.001), pulmonary disease (21.5% vs. 12.4%, p < 0.001) and anemia (43.5% vs.37.9%, p = 0.005), whereas only dilated cardiomyopathy (25.8% vs. 7.1%, p < 0.001) was more common in the Chinese cohort.

**Fig. 2:**
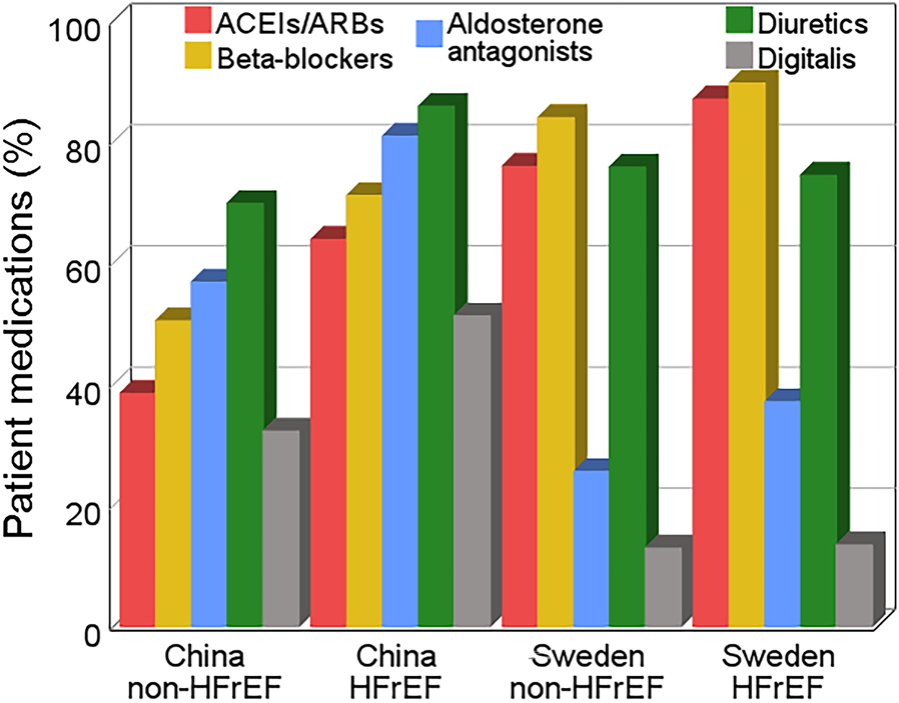
Guideline-directed medical therapy in the two cohorts

The use of angiotensin converting enzyme inhibitors/angiotensin receptor blockers (ACEIs/ARBs) (48.8% vs. 80.8%, p < 0.001) and beta-blockers (BBs) (58.8% vs. 86.5%, p < 0.001) was lower, whereas that of mineralcorticoid receptor antagonists (MRAs) (66.6% vs. 30.6%, p < 0.001) and digitalis (39.9% vs. 13.1%, p < 0.001) was higher in the Chinese cohort than in the Swedish cohort. As shown in Fig. 2, the HFrEF group was more likely to receive medical therapy than the non-HFrEF group in both cohorts. In patients with HFrEF, 20.4% of the Chinese patients and 64.6% of the Swedish patients were on 50% guideline-directed medical therapy (GDMT) target ACEIs/ARBs dose; 6.4% of the Chinese patients and 56.9% of the Swedish patients were on 50% GDMT target BBs dose; 79.9% of the Chinese patients and 32.3% of the Swedish patients were on 50% GDMT target MRAs dose (Additional file 1: Fig. S1). The reasons for no use of selected GDMT in each cohort were demonstrated in Additional file 1: Fig. S2.

### Age stratified clinical characteristics and 6-month prognosis

The 6-month crude all-cause mortality was lower in the Chinese cohort than in the Swedish cohort (9.8% vs. 20.7%, p < 0.001). In the age group of age < 50 years, 50–74 years and ≥ 75 years, the mortality was 10.7%, 8.7% and 9.3% respectively in Chinese patients, in contrast to 0, 11.0% and 27.2% in Swedish patients.

There were 205 out of 949 Chinese patients (21.6%) and 69 out of 1639 Swedish patients (4.2%) in the age group < 50 years. The three most common comorbidities were dilated cardiomyopathy (40.5%), valvular disease (34.1%), atrial fibrillation/flutter (33.2%) in the Chinese cohort. HFrEF was noted in 53.7% of the patients and the median level of NT-proBNP was 3622 (IQR 1730–6793) pg/mL. The three most common comorbidities of the Swedish cohort included valvular disease (33.3%), hypertension (29.0%) and dilated cardiomyopathy (29.0%). HFrEF was diagnosed in 44.9% of the patients and the median level of NT-proBNP was 1100 (222–3699) pg/mL.

There were 194 (20.4%) Chinese patients and 1026 (62.6%) Swedish patients in the age group ≥ 75 years. Median age of this Chinese cohort was 79 (IQR 76–82) years. The five most prevalent comorbidities were hypertension (64.4%), renal dysfunction (60.3%), anemia (55.7%), diabetes (43.8%) and atrial fibrillation/flutter (40.7%). In this cohort 21.6% of Chinese patients had HFrEF and the median value of NT-proBNP was 3665 (IQR 1436–8742) pg/mL. In the Swedish cohort, median age of the patients was 84 (IQR 80–89) years, and approximately half of the deceased were older than 80 years. The five most prevalent comorbidities were hypertension (71.7%), renal dysfunction (65.9%), AF (63.7%), valvular disease (46.7%) and anemia (49.5%). 59.1% of Swedish patients had HFrEF and the median value of NT-proBNP was 4978 (IQR 2209–9741) pg/mL.

**Fig. 3:**
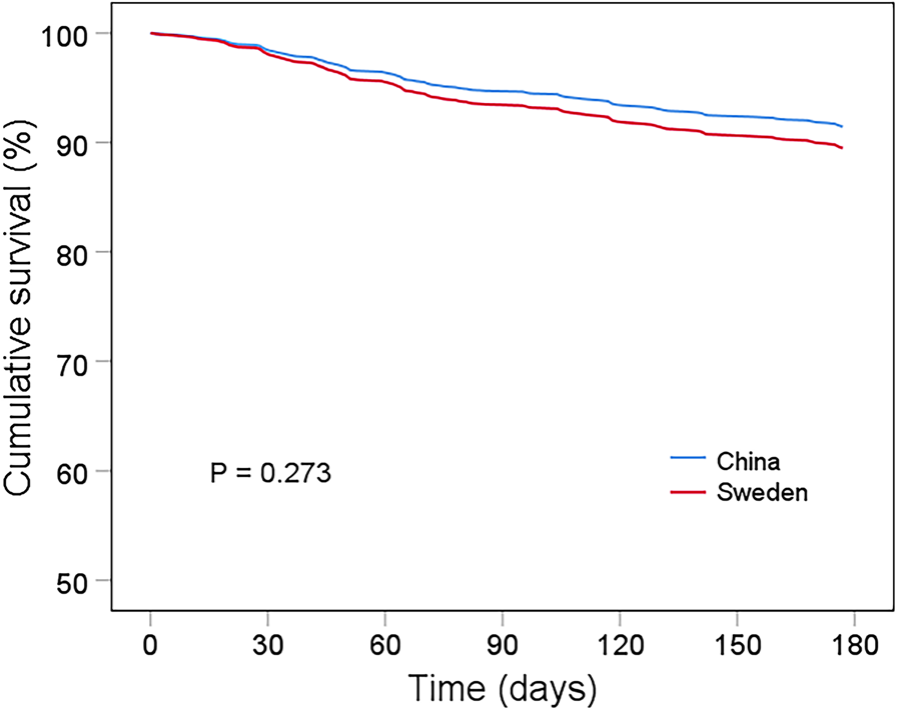
Age- and sex-adjusted Kaplan–Meier survival curves in patients aged 50–74 years

There were 550 (58.0%) Chinese patients and 554 (33.2%) Swedish patients in the age group 50–74 years. Figure 3 illustrates the age- and sex-adjusted Cox regression survival analysis between the two cohorts (HR 0.80 [95% CI 0.55–1.19], p = 0.273). Differences between the two study populations were obvious in terms of demographics, medical history, physical examination, lab tests and treatment, as shown in Table 2. The Swedish cohort had more patients with ischemic heart disease or hypertension, as well as more patients treated with ACEIs/ARBs or beta-blockers. Higher systolic and diastolic blood pressure with lower resting heart rate were also present in this cohort.

**Table 2:** Baseline characteristics of patients aged 50–74 years in the two cohorts

Variables	Chinese cohort (N = 550)	Swedish cohort (N = 544)	P value
*Demographics*			
Age, years*			
Mean (SD)	63.6 ± 6.8	65.3 ± 6.4	** < 0.001**
Median (IQR)	64 (59–69)	67 (60–70)	** < 0.001**
Female, n (%)*	251 (45.6)	152 (27.9)	** < 0.001**
*Clinical history, n (%)*			
Ischemic heart disease*	99 (18.0)	177 (32.5)	** < 0.001**
Dilated cardiomyopathy	138 (25.1)	66 (12.1)	** < 0.001**
Hypertension	190 (34.5)	294 (54.0)	** < 0.001**
Valvular disease	183 (33.3)	169 (31.1)	0.435
Atrial fibrillation/ flutter*	229 (41.6)	241 (44.3)	0.373
Diabetes mellitus*	172 (31.3)	187 (34.4)	0.275
Pulmonary disease	66 (12.0)	115 (21.1)	** < 0.001**
Anemia*	199 (36.2)	191 (35.1)	0.711
*Physical/laboratory*			
SBP, mmHg*	114.0 ± 15.3	123.0 ± 19.1	** < 0.001**
DBP, mmHg	69.6 ± 9.9	72.6 ± 10.7	** < 0.001**
Heart rate, beats/min*	82.8 ± 19.9	70.9 ± 13.4	** < 0.001**
Hemoglobin, g/L	130.4 ± 24.0	132.4 ± 18.9	0.271
eGFR, ml/min/1.73m^2^*	71.7 ± 25.0	67.8 ± 25.2	**0.027**
NT-proBNP,	3121	3000	0.119
Median (IQR), pg/mL*	(1418–7693)	(1096–7296)	
LVEF < 40%*	235 (42.7)	272 (50.0)	**0.016**
QRS duration ≥ 120 ms, n (%)*	174 (31.6)	183 (33.6)	0.48
LBBB, n (%)	110 (20.0)	54 (9.9)	** < 0.001**
*Medication, n (%)*			
ACEIs / ARBs*	275 (50.0)	492 (90.4)	** < 0.001**
Beta-blockers*	333 (60.5)	503 (92.5)	** < 0.001**
Aldosterone antagonists*	377 (68.5)	196 (36.0)	** < 0.001**
Diuretics	411 (74.7)	350 (64.3)	** < 0.001**
Digitalis	224 (40.7)	71 (13.1)	** < 0.001**
*Device therapy, n (%)*			
Pacemaker	18 (3.3)	48 (8.8)	** < 0.001**
CRT-P/D	42 (7.6)	18 (3.3)	**0.002**
ICD	31 (5.6)	17 (3.1)	**0.043**

Abbreviations as in Table 1

Variables labelled with asterisk (*) were included in Cox regression analysis

### Predictors of 6-month mortality

In the age group of 50–74 years, multivariate Cox regression model was established for predictors of 6-month all-cause mortality in the Chinese cohort and the Swedish cohort, respectively. By including common clinical parameters as shown in Table 3, higher NT-proBNP and lower eGFR were independent predictors of higher mortality in both cohorts. Besides, older age, lower systolic blood pressure and prolonged QRS duration were associated with increased death rate in the Swedish cohort.

**Table 3:** Predictors of 6-month mortality in patients aged 50–74 years

Variables	Chinese cohort	P value	Swedish cohort	P value
	HR (95% CI)		HR (95% CI)	
Age	0.957 (0.914–1.003)	0.066	1.110 (1.047–1.177)	**< 0.001**
Female	1.079 (0.572–2.035)	0.815	1.565 (0.869–2.816)	0.135
SBP (per 10 mmHg)	0.876 (0.717–1.071)	0.198	0.818 (0.699–0.956)	**0.012**
Heart rate (per 10 beats/min)	0.983 (0.834–1.160)	0.841	1.211 (0.992–1.479)	0.060
Ischemic etiology	1.248 (0.553–2.813)	0.594	1.002 (0.558–1.799)	0.994
Atrial fibrillation/flutter	1.278 (0.696–2.348)	0.428	0.917 (0.515–1.633)	0.769
Diabetes mellitus	0.936 (0.489–1.791)	0.841	1.005 (0.573–1.763)	0.987
Anemia	1.209 (0.656–2.228)	0.543	1.657 (0.944–2.908)	0.079
QRS ≥ 120 ms	1.297 (0.658–2.558)	0.452	1.854 (1.038–3.310)	**0.037**
NT-proBNP (per 100 pg/mL)	1.006 (1.003-1.008)	**< 0.001**	1.003 (1.000-1.007)	**0.031**
LVEF < 40%	1.083 (0.540–2.173)	0.822	1.075 (0.598–1.933)	0.809
eGFR	0.986 (0.973–0.999)	**0.033**	0.988 (0.976–0.999)	**0.043**
ACEIs/ARBs	0.935 (0.485–1.803)	0.841	1.172 (0.475–2.888)	0.730
Beta-blockers	0.537 (0.284–1.016)	0.056	1.007 (0.368–2.753)	0.990
Aldosterone antagonists	0.874 (0.449–1.702)	0.692	0.570 (0.308–1.054)	0.073

Abbreviations as in Table 1

Values in bold indicate P value < 0.05

## Discussion

This patient-level comparison of hospitalized HF cohorts in China and Sweden revealed several main findings: (a) Clinical phenotype of HF population was distinct from each other. Swedish patients were 15 years older with more comorbidities while Chinese patients had lower blood pressure and NT-proBNP level; (b) Utilization of guideline-directed medical therapy was more frequent in Swedish patients with more prescription of ACEIs/ARBs and/or beta blockers but more Chinese patients were given MRAs and/or digitalis; (c) Overall 6-month mortality was higher in the Swedish cohort because of higher age, while the mortality was similar in patients of 50–75 years; (d) NT-proBNP and eGFR were independent predictors of 6-month mortality in both cohorts.

Comparison of HF patients from developing versus developed countries has been described by few studies [8–10]. Almost exclusively, HF patients from Asian areas or low- and middle-income countries were generally younger than those from Europe or high-income countries as observed in the current study. Apart from the age gap in this study, the Swedish cohort seemed to have “sicker” patients with heavier comorbidity burdens and more death than the Chinese cohort and the proportion of patients with dilated cardiomyopathy also varied enormously. It may indicate not only aging population in Sweden, but also different healthcare system between the two countries where Sweden has stricter referral criteria to tertiary care and higher thresholds of hospitalization [4, 29]. On the other hand, it may be explained by that more younger patients with dilated cardiomyopathy in China tend to seek medical service in the tertiary hospital but those patients with worsening HF are not routinely hospitalized in the tertiary settings, particularly the elderly with lower standard of living and poorer educational level from remote rural areas [30].

Sweden is one of the leading countries that early investigated and implemented neurohormonal suppression therapy in HF patients. Use of both renin-angiotensin system (RAS) antagonists and beta blockers in patients with EF < 30% were approximately 90% already in 2003 and remained constant over time [31]. In contrast, previous studies showed relatively worse performance in prescribing these medicines and guideline adherence when China was compared with European countries [17, 32–34], and the treatment with RAS antagonists and beta blockers never exceeded 80% [35]. Even if the underuse of ACEI/ARBs and beta blockers in the Chinese cohort of this study could partially be explained by contraindicated blood pressure in some patients (the Chinese cohort presented lower BP), there is still room for improvement in utilization of these drugs. On the contrary, more prescriptions of digitalis in the Chinese cohort may reflect the difference in the clinical practice of its good acceptance by Chinese doctors in terms of symptom relief and low cost, which might be also associated with reimbursement patterns of the countries.

Given unmatched age of the two populations, age-stratified analysis helped us to understand that senior patients with multiple diseases contributed to higher mortality in the Swedish cohort. Within the age group < 50 or ≥ 75 years, it is rational not to perform further comparison except descriptive analysis because of so big difference in sample size. Interestingly, similar to the China-HF registry that showed higher in-hospital mortality in younger (< 40 years) than older adults [17], the current study observed higher 6-month mortality in younger age group (< 50 years) of the Chinese cohort. It is in contrast to the established concept that death increases with age in HF population; but might be explained by an underestimated mortality in the senior age group due to fewer referrals of seriously ill old patients to tertiary hospitals [30]. In the Swedish cohort, there was no death in the youngest age group, < 50 years, that may be only attributed to the limited number of patients. A previous study reported an unadjusted 6-month mortality of about 10% among those aged ≤ 54 years and about 20% among those ≥ 55 years during 2002–2006 [36].

What is most interesting is when both cohorts were compared when they were in the similar age, aged 50–74 years, even if the mean age was 1.7 years higher in the Swedish cohort. In this case, the mortality was similar between cohorts though clinical characteristics and heart failure therapy differed. The question is why different phenotypes and different therapy were associated with similar mortality in two cohort in similar age? One possible explanation is that factors affecting survival were balanced between cohorts. For instance, in the Chinese cohort dilated cardiomyopathy was more frequent than that in the Swedish cohort, and vice versa for ischemic heart disease. It is known that dilated cardiomyopathy has better prognosis than ischemic heart disease. In the meantime, Swedish patients received optimal treatment with ACEIs/ARBs and beta blockers. In our study, a similar mortality was associated with similar NT-proBNP levels between cohorts. In accordance with previous reports [37, 38], increased NT-proBNP and decreased eGFR, common risk factors in both cohorts, independently predicted 6-month mortality. Nevertheless, some predictors tested in previous studies such as age and systolic blood pressure were only significant in the Swedish cohort, the reasons for that were not well illustrated.

This study had several limitations. First, a pooled data analysis was not equal to a pre-defined multi-center prospective study, though the difference was minimized by including those parameters with unified definitions acceptable to both sides. Second, different age distribution of the entire two cohorts and unmatched patient numbers in two age groups (< 50 years and ≥ 75 years) resulted in that head-to-head comparison was only conducted in the age group of 50–74 years. Third, the information obtained from patients enrolled in single-center registries may not be generalized and representative of the Chinese and Swedish HF patients who were admitted to tertiary referral hospitals. Besides, the present study focused on the only outcome of 6-month all-cause mortality but other concerned outcomes such as cardiovascular mortality, rehospitalization were lacking. Some previous reported important variables associated with the prognosis of HF such as New York Heart Association functional class, body mass index and coronary revascularization failed to be included into analysis because of their substantial missing values (> 25%) and also some parameters have not been collected either in Chinese or Swedish study when the registry initiated. Those factors which were not adjusted for might affect the difference in mortality between the cohorts.

## Conclusions

This study investigated patient-level comparison from single-center HF registries in China and Sweden, which revealed different clinical phenotype but similar prognosis between the two cohorts beyond age.

## Supplementary Information

**Additional file 1.** **Fig. S1.** Patients with HFrEF on 50% GDMT target dose. **Fig. S2.** Reasons for no use of selected GDMT (**A** ACEIs/ARBs; **B** beta-blockers) in patients with HFrEF.

## Data Availability

The datasets used and/or analyzed during the study is available from the corresponding author on reasonable request.
