# Supplementary material for: Patient-level comparison of heart failure patients in clinical phenotype and prognosis from China and Sweden
Source: BMC Cardiovasc Disord. 2022 Mar 8;22:91. doi: 10.1186/s12872-022-02540-w (PMC8903728; doi:10.1186/s12872-022-02540-w)
Supplement: Supplementary file 1 — Additional file 1. Fig. S1. Patients with HFrEF on 50% GDMT target dose. Fig. S2. Reasons for no use of selected GDMT (A ACEIs/ARBs; B beta-blockers) in patients with HFrEF. [file 12872_2022_2540_MOESM1_ESM.docx]

**Supplementary Material**

**Supplementary Figure**

Fig S1. Patients with HFrEF on 50% GDMT target dose

Fig S2. Reasons for no use of selected GDMT (A. ACEIs/ARBs; B. beta-blockers) in patients with HFrEF


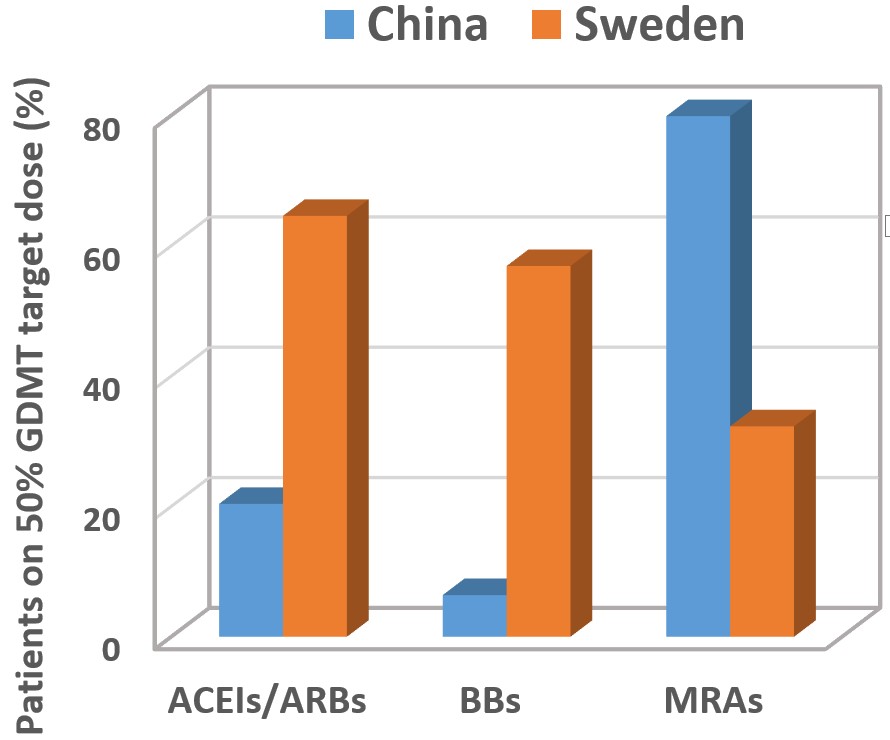


Fig S1. Patients with HFrEF on 50% GDMT target dose


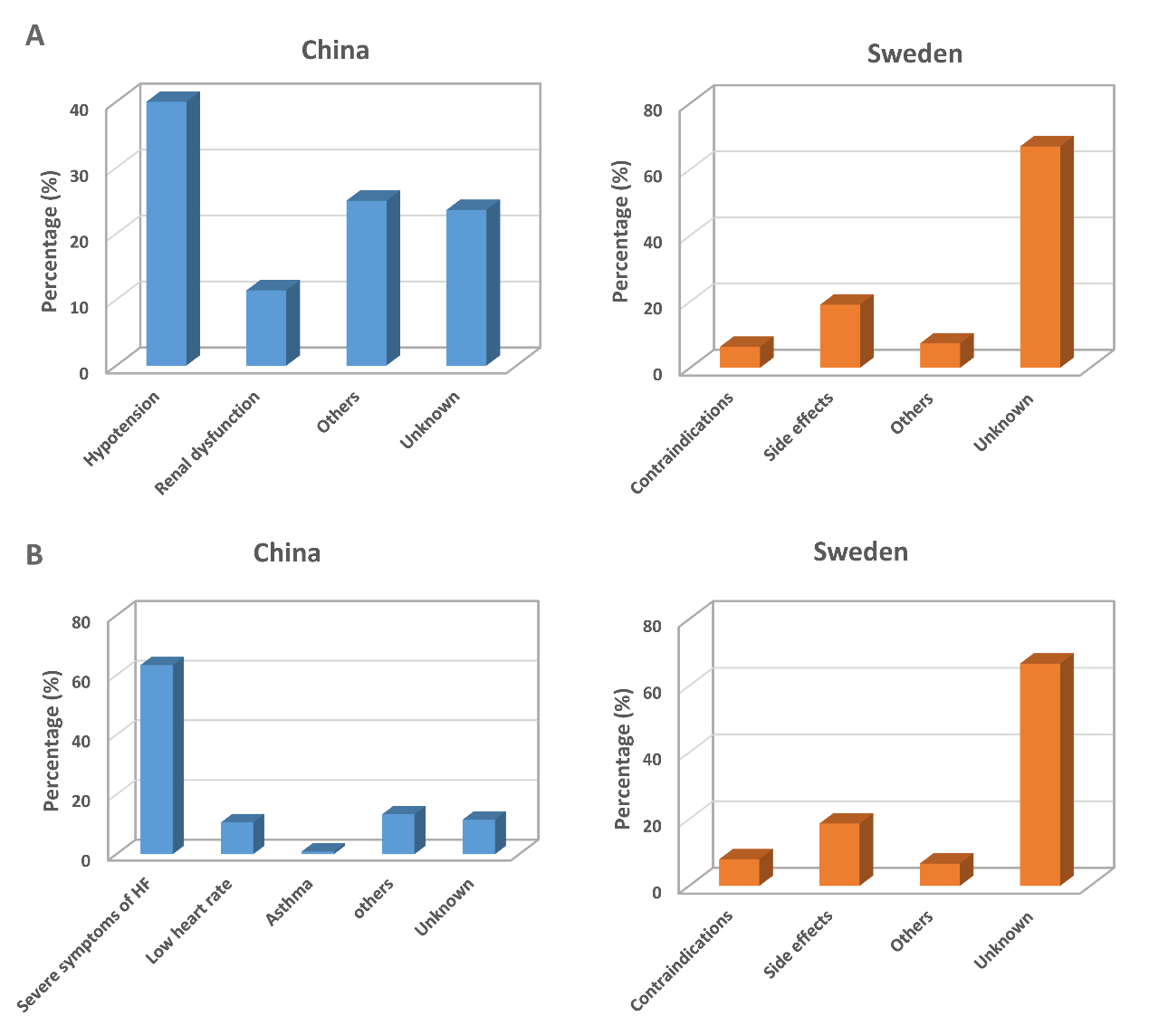


Fig S2. Reasons for no use of selected GDMT (A. ACEIs/ARBs; B. beta-blockers) in patients with HFrEF
